# Predicting diagnostic outcome in adult autism spectrum disorder using the autism diagnostic observation schedule, second edition

**DOI:** 10.1186/s12888-020-03028-7

**Published:** 2021-01-10

**Authors:** Marios Adamou, Sarah L. Jones, Stephanie Wetherhill

**Affiliations:** 1 University of Hudderfield, Huddersfield, UK; 2 South West Yorkshire Partnership NHS Foundation Trust, Wakefield, UK; 3 Manygates Clinic, Belle Isle Health Park, Portobello Road, Wakefield, WF1 5PN UK; 4 Leeds Community Healthcare, Leeds, UK

**Keywords:** ASD, Adult ASD, Autism, ADOS, ADOS-2, Diagnostic assessment, Multidisciplinary

## Abstract

**Background:**

The Autism Diagnostic Observation Schedule, Second Edition (ADOS-2) module four assessment for diagnosing autism spectrum disorder in adults has shown good sensitivity and specificity in research settings.

**Method:**

This study aimed to evaluate the predictive accuracy of the ADOS-2 module four by investigating the components of the assessment, in relation to diagnostic outcome in a clinical setting. Data from 88 service users referred to a Specialist Adult Autism Service was explored.

**Results:**

ADOS-2 scores failed to predict the diagnostic outcome (overall sensitivity = 92%, specificity = 57%). Interestingly, scores from the ‘restricted interests’ component of the ADOS-2 have the potential to predict diagnostic outcome, despite this domain not been included in the scoring algorithm.

**Conclusions:**

Based on our findings, we recommend clinicians are cautious when interpreting results of the ADOS-2 assessment.

Autism spectrum disorder (ASD) is a neurodevelopmental disorder characterised by pervasive difficulties in reciprocal social interaction, alongside the presence of strict repetitive interests and behaviours [1]. Whilst much research in ASD focuses on the developmental period, it is recognised that ASD is a lifelong condition [2–6], which is sometimes not detected clinically until later life. This delay in recognition may be explained by the observation that the ASD phenotype presents with a range of severities, language ability and intellects [7], but also because masking behaviour [8, 9] or compensation strategies may not bring out sufficient impairment [10] to lead a person to a clinical assessment.

Diagnosing ASD in adulthood can be difficult for a number of reasons: First, it is resource intensive due to the amount of information which needs to be collected, ideally from a variety of sources. If input from a parent or caregiver is not accessible, it can be challenging to build an accurate account of the neurodevelopmental period, as self-insight from the patient may be unreliable [11, 12]. Second, it requires a high level of specialisation by professionals who are not always available for service. Also, presentation of symptoms can greatly overlap with other disorders, specifically, negative symptoms of schizophrenia [13, 14], as well as other psychiatric co-morbidities [15], rendering the diagnostic picture complex [16]. This requires trained and experienced specialists working together in a multidisciplinary environment to ensure accuracy of diagnosis [16]. Taking heed from recent NICE guidelines for ASD in adults [17], diagnosis of ASD in adulthood is reached on a consensus of expert opinion made by observations from multidisciplinary assessments, including detailed developmental history taking (if available), current behavioural factors, and cognitive abilities [7].

One of the tools used in diagnosing ASD is the Autism Diagnosis Observation Schedule, second edition (ADOS-2) [18]. The ADOS-2 (all variations) is an interactive, standardized assessment designed for supporting the diagnostic process [19] and is considered as a ‘gold-standard’ in any diagnostic protocol [16, 20].

The ADOS-2 consists of four modules which evaluate reciprocal social communication, reciprocal interests, imagination and restricted interests [21] mapping the domains of the DSM-IV [22]. Individuals are evaluated by only one of the modules, which is chosen by the administrator, based on the individual’s expressive language ability and chronological age [18]. For instance, modules one to three are used for assessment of children and adolescents of varying language levels, module four is used to assess adolescents and adults with fluent language ability. The coded scoring system creates an algorithm which consists of communication, reciprocal interests and imagination score. The idea is that the ADOS allows the administrator to gather relevant information in a systematic manner, in order to produce a notion of objectivity to aid diagnosis [7]. In terms of clinical administration, the ADOS-2 is often employed in conjunction with the Autism Diagnostic Interview-Revised (ADI-R) [19]. The ADI-R is an administrator-based semi-structured interview designed to make available a developmental history and picture of current functioning for individuals with a mental age of 2 years or above [7]. Employed together, the ADOS-2 and ADI-R assessments are considered the most useful diagnostic aid for ASD [21, 23, 24].

The ADOS (1), and revised ADOS-2 [18], have received considerable research attention. A fair assessment would suggest that the variations of the ADOS have shown to be useful and reliable in their assessment of ASD in childhood and adolescent studies [19, 25–29]. For instance, de Bildt et al. [25] found that together with the ADI-R, in a sample of 184 children, the ADOS-Generic (G) accurately identified ASD and Pervasive Developmental Disorder (PDD) as per the DSM-IV criteria. Mazefsky and Oswald [26] also explored the diagnostic utility of the ADOS-G in a sample of children, finding that together with the ADI-R, the ADOS-G was in agreement with clinical decision making 75% of the time. Similarly, Risi et al. [28] employed a childhood sample to explore the use of ADOS assessment, concluding that the ADOS, together with the ADI-R makes an objective, consistent contribution to diagnostic decision making. Further, Gotham et al. [27] in a large sample of children aged 2 to 16 years, found the ADOS was able to approximate ASD severity, concluding that the assessment is useful in clinical settings. In terms of sensitivity, Kamp-Becker et al. [29] also explored the revised version of the ADOS in a paediatric sample, concluding that it is a valid and reliable measure of ASD, with good sensitivity for subtle ASD, and high functioning ASD. In addition, Kamp-Becker et al. [19] investigated the ability of module three in high-functioning ASD in children aged 4 to 16 years with full scale IQ above 70, also concluding that the ADOS operates with good sensitivity. On the other hand, Molloy et al. [16] compared ADOS classification to final diagnosis by a multidisciplinary team, in a sample of 584 children, using modules one, two, and three. Interestingly, they found that using numerical scores alone, resulted in false positives, therefore they recommend clinicians use the qualitative information gathered during the ADOS assessment along with scoring to gather a clearer clinical picture, rather than relying on scores alone, which they suggest, fail to provide a reliable formulation.

Studies exploring the usefulness of ADOS-2 module four, (for adult populations) are scarce compared to those investigating ADOS in relation to childhood cohorts. In the original paper assessing the validity of module four, the study employed a small sample of young adults with ASD (*n* = 16), PDD-not otherwise specified (NOS) (*n* = 16), and other diagnoses (*n* = 15). The results suggested that module four can be used effectively to distinguish between ASD and neurotypical profiles, but could not *as easily* distinguish between ASD and PDD-NOS [30]. Of the limited knowledge gathered since the publication of the original paper, Bastiaansen et al. [10] explored high-functioning ASD in a male only adult sample, in comparison to three other groups; schizophrenia, psychopathology and typical development. The authors conclude that module four was a good predictor of distinguishing between ASD and the other conditions, however less able to discriminate between ASD and negative symptoms of schizophrenia due to symptom overlap. A revision to module four was made in 2014 [31] to bring module four in line with modules one to three to improve diagnostic validity. The revised algorithm included greater consistency with DSM-5 criteria and saw an improved sensitivity of 90% and maintained specificity at 82% [31]. de Bildt [32] examined the ability of the ADOS-2, module four, in a male only sample, to distinguish between ASD, schizophrenia, psychopathology, and controls. They concluded that module four could discriminate between neurotypical profiles, ASD, and psychopathy; however, it was not able to discriminate schizophrenia from ASD as easily. Langmann et al. [33] investigated module four of the revised version of the ADOS assessment, compared to the original algorithm, in a clinical sample consisting of adolescents and adults with high functioning ASD. They found support for both the original and revised version of the ADOS-2, concluding good sensitivity, and support the utility of ADOS-2 in clinical decision making. Further, Fusar-Poli et al. [21] evaluated the sensitivity and specificity of the ADOS and ADI-R in a sample of 113 adults with an IQ of 70 or above. They suggest that module four may cautiously boast accuracy of diagnosis, however, only if the individual is without intellectual disability.

Naturally, diagnostic tests are not designed to be used in isolation. Rather, they should be used as estimates of probability and curtail diagnostic uncertainty by aiding clinical formulation [34]. Certainly, tests that are validated by sensitivity and specificity are susceptible to biases that effect test efficacy, such as variability across populations and severity of condition [35, 36], which is likely to be a factor in our observation that we often see disparity between ADOS-2 classification and overall diagnostic outcome in our clinic. Based on this, we take a novel approach to the investigation of the ADOS-2 module four assessment. We were interested to measure sensitivity and specificity in our sample. We hypothesised that there would be disparity between ADOS-2 threshold score and diagnostic formulation. Also, we wanted to explore if *specific* domains of the assessment were predictive of the final diagnostic outcome, particularly considering that ASD presentation can be varied in severity and symptomology [7]. Data for this study was gathered retrospectively from a clinical sample of adult mental health service users referred for possible ASD between 2017 and 2018, to the Adult ADHD and Autism Service, South West Yorkshire Partnership NHS Foundation Trust.

## Methods

### Participants

The sample employed 88 adults referred for ASD assessment to Specialist Adult ADHD and Autism Service, South West Yorkshire Partnership NHS Foundation Trust, in the South and West Yorkshire geographical area of the UK, between 2017 and 2018. The Adult ADHD and Autism Service is a specialist service in diagnosing ADHD and Autism in adulthood. Patients without intellectual disability are referred to the service by health care professionals, whom deem it appropriate based on history and current difficulties. Inclusion criteria dictated that participants were over the age of 18 years (no cut-off), had a good comprehension of the English language, and IQ within normal range i.e. > 70. Patients accessing the service are routinely informed that their data can be used for research purposes and have the opportunity to opt-out. For this project, the need for ethics approval was waived by SWYPFT Research and Development Department as the data was gathered retrospectively and was collected as part of the clinical operations of the service. The SWYPFT Caldicott Guardian endorsed access to data following Caldicott Principles. Data was gathered from electronic records. Gender was measured by asking the participants to report male, female or prefer not to say. The sample consisted of 58 (65.9%) males, 30 (34.1%) females, with no participants choosing not to disclose gender. Mean age was 34.1 years (*SD*± 12.9).

### Assessment

The ADOS-2, module four (for adolescents and adults with fluent language ability) [18] is principally a semi-structured interview, involving tasks which facilitate interaction between the interviewee and the administrator, which takes on average 60 min to complete. Protocol guides that the administrator is directed by a booklet which is used to provide structure, take notes, and subsequently use the coding section (directly after the assessment) along with the algorithm, to formulate a score based on observations during the assessment (more information on this process can be obtained here [18]). The ADOS-2 assessment assumes features of ASD are likely to be present if the scoring cut-off is exceeded. Lord et al. [18] advises that cut-off values for ASD in the communication component is a score of two or above, the social component is a score of four or above, and the communication + social component is a score of seven or above. Imagination, Stereotyped Behaviours, and Restricted Interests components, which are also quantified, are not considered in the final scoring as it was suggested that the narrow window of the assessment may not be sufficient to elicit such behaviours [37].

### Procedure

Data was collected as part of the clinical evaluation of adults referred to a Specialist Adult ADHD and Autism Service. The Service consists of a highly specialist multidisciplinary team. The professionals who administer the ADOS-2 have undertaken formal ADOS-2 training, undergo yearly cross validation, hold six-monthly inter-rater reliability meetings, and are competent in the knowledge of administering and scoring the assessment. The ADOS-2 indicative formulation is based on interpretation of the assessment and scoring. The assessment is a part of the diagnostic process, but by no means determines the diagnostic outcome; this is only done after a multidisciplinary decision making which includes other sources of information. This includes information through history taking, mental state examination, observations of the interactions during the assessment, assessments of daily functioning and from use of other standardised tools such as the ADI-R.

### Statistical analysis

Binomial logistic regression was employed, to investigate the likelihood of a service user receiving a positive or negative ASD diagnostic outcome. Employing ADOS-2 (module four) scores as independent variables, we explored the predictive value of the outcome variable, which was subsequent confirmed diagnosis of ASD based on expert clinical judgements. Analysis was performed to ascertain the usefulness of scores of the Communication, Reciprocal interaction, Imagination, and Restricted Interests domains, dependent on the likelihood that patients received a diagnosis of ASD. Linearity of the continuous variables with respect to the logit of the dependent variable was assessed via the Box and Tidwell [38] procedure. A Bonferroni correction was applied using all terms in the model [8] resulting in statistical significance being accepted when *p* < 0.00625. Based on this assessment, all continuous independent variables were found to be linearly related to the logit of the dependent variable, necessary for the analysis. In terms of outliers, there were no data points more than two. The Hosmer and Lemeshow goodness of fit test revealed the model was a good fit at predicting the categorical outcome (*ns*).

## Results

The study included 88 participants, there were five cases of missing data, and therefore 94.3% of the sample was included in the analysis. There were no differences found for age or sex between ASD and non-ASD outcome groups (*ns*). Overall, 26 patients (29.5%) received a final diagnostic outcome of ASD by clinical consensus as described above. Those who received a clinical diagnosis of ASD scored greater on the ADOS (*M* = 12.85, *SD±* = 3.7) than those who did not (*M* = 8.26, *SD±* = 5.2) (*p*< 0.01). For males, the diagnostic rate was 32.8% and for females it was 23.3%. In terms of meeting the threshold score for ADOS, 48 patients (54.5%) scored above the diagnostic threshold (*Median* = 9, *Range* = 24). Subgroup analysis showed that males tended to score higher on the ADOS (*Median* = 11, *Range* = 24) than females (*Median* = 9, *Range* = 17), but not at a significant level (*ns*). Comparison of ADOS classification comparative to clinical diagnosis can be found in Table 1.

**Table 1:** Comparison of ADOS classification comparative to clinical diagnosis

Classified (ADOS)	**48 (57.8%)**	Diagnosed (Clinically)	**24 (50%)**
Non classified (ADOS)	**35 (42.2%)**	Diagnosed (Clinically)	**2 (5.7%)**
Classified (ADOS)	**48 (57.8%)**	Non diagnosed (Clinically)	**24 (50%)**
Non classified (ADOS)	**35 (42.2%)**	Non diagnosed (Clinically)	**33 (94.3%)**

### Sensitivity, specificity and predictive values

The ADOS-2 module four, demonstrated 92% sensitivity at detecting the presence of ASD in those who received a clinical diagnosis, however only 57% specificity at detecting the absence of ASD in those who did not receive a clinical diagnosis. Positive predictive value (PPV) determined that if a patient scored above the ADOS cut-off, they have a 50% chance of receiving a clinical diagnosis. Negative predictive value (NPV) determined that 94% of those who did not score above the threshold did not receive a clinical diagnosis.

Mean scores for individual components of the ADOS-2 assessment can be found in Table 2. In order to explore the predictive value of the individual components of the ADOS-2 module four, regression analysis was conducted. The area under the ROC curve was .792, 95% *CI* [.696, .887], which is an acceptable level of discrimination according to Hosmer & Lemeshow [39]. The logistic regression model was statistically significant **χ**^**2**^**(4) = 16.262,** ***p*** **= .003** (Table 3). The model explained 25% (Nagelkerke *R*^*2*^) of the variance in diagnostic outcome and correctly classified 72.3% of cases. Of the four predictor variables, ‘restricted interests’ was most closely related to predicting the final diagnostic decision.

**Table 2:** Mean scores for ADOS-2 sub-domains in outcome groups

	M (SD±)	
	ASD	Non-ASD
Communication	**3.69 (1.6)**	**5.53 (14.1)**
Reciprocal interests	**6.96 (2.5)**	**4.69 (3.4)**
Imagination	**1.08 (.69)**	**.76 (.70)**
Restricted interests	**1.15 (1.3)**	**.60 (0.8)**

**Table 3:** Logistic regression predicting likelihood of ASD diagnosis based on ADOS-2 module four domains

	***B***	SE	Wald	***df***	***p***	Exp(B)	95% CI for Exp(B)	
							Lower	Upper
**Communication**	.181	.177	1.044	1	.307	1.198	.847	1.694
**Reciprocal interests**	.173	.110	2.494	1	.114	1.189	.959	1.474
**Imagination**	.175	.403	.189	1	.664	1.191	.541	2.623
**Restricted interests**	.558	.261	4.570	1	.033	1.747	1.047	2.913
**Constant**	−2.971	.731	16.534	1	.000	.051		

## Discussion

With a growing population, the demand for ASD diagnosis is increasing. Expert opinion bids that formulating an ASD diagnosis in adulthood is often complex, as clinical presentation is varied. It becomes especially difficult if retrospective developmental information is unavailable to the diagnostic process [10]. Variations of the ADOS assessment have become an integral part of the diagnostic protocol in both childhood and adulthood cohorts, providing important quantified information as an element of an objectification. Thus, investigation into the usefulness and accuracy of commonly used assessments is essential.

Whilst we found that those patients who went on to receive a diagnosis scored greater overall, our interest here was the predictive efficacy of the scores of the individual ADOS-2 domains, in relation to final diagnostic formulation by an expert multidisciplinary team, in a sample of adults referred to a specialist Service for possible ASD. Previous research advocates that support for the inclusion of the ADOS assessment as part of the diagnostic formulation in both childhood and adulthood is good, albeit for the issues previously discussed [19, 25–29, 40, 41]. Results here demonstrated relatively high sensitivity (0.92) compared to other studies [31, 32, 42]. However, results here also demonstrate low specificity (0.57), the lowest of previous reports [31, 32, 42]. To further this investigation, we took a novel approach and examined the individual domains of the ADOS-2, module four assessment in order to explore if there are individual elements of the assessment that held predictive power for final diagnostic outcome. Here, we found that the restricted interests domain showed the potential to predict final diagnostic outcome (*p*< 0.03, *trend* when adjusted for multiple comparisons). However, other domains (communication, reciprocal interests and imagination) failed to share a relationship with whether or not a person was ultimately diagnosed with ASD after expert formulation in our sample.

Restricted interests in ASD are heterogeneous, consisting of intensive repetitive behaviours, such as narrow interests, motor mannerisms, or cognitively mediated symptoms such as rituals or rigid insistence on specific environmental factors or routine [43, 44]. Consequently, living with such behaviours can often significantly impact daily living. There is also neurological evidence to suggest restricted interests particular to ASD, parallel enhanced insula and anterior cingulate response to repetitive behaviours [45], albeit research is as yet in its infancy [46].

The finding that restricted interests was most closely related to diagnostic outcome is somewhat surprising, especially when considering that the restricted interests domain is quantified, but not designed to be a part of the ADOS-2, module four, diagnostic algorithm, despite it being one of the core features of ASD [46]. Instead, only scores on social interaction and communication make up the final score. It has been suggested by the authors of the ADOS assessment that elements of restricted interests and repetitive behaviours may not be sufficiently reliable to be included in the overall score, in that the nature of the assessment may not trigger a true account of these behaviours [7, 31]. Interestingly, in the revised version of the ADOS assessments, the authors have made way for a Restricted, Repetitive Behaviour (RRB) domain, which is now included in the algorithm in modules one, two, and three [31]. Yet, restricted interests has not been implemented in module four. Reasons for this are not clear, although it has been suggested that including a restricted behaviours element to the scoring algorithm would reduce sensitivity of the assessment, as some individuals with ASD will show reduced levels of this behaviour. Further to this, there is some evidence that restricted interests in ASD cohorts are not as apparent in later life, as they are for younger groups [43, 46, 47]. However, the results presented here suggest restricted interests should perhaps be considered a part of the scoring, as the presence of restricted interests was closely related to diagnostic outcome. Indeed, revisions to module four have found that the addition of RRBs in the overall score equal to that of modules 1–3, improves sensitivity and specificty [31]. Further research with larger and more diverse samples is required to replicate the trends found in this study, however, by building on this narrative, possible revisions to the ADOS-2 module four may be considered.

The original study of module four was a relatively small, lab based study, with a diverse sample in terms of pathology [18]. It has been suggested, and we would concur, that findings produced from studies of this type, should be interpreted within the context of the study protocol [16]. In that, the clinical populations for which the ADOS is employed, is different from the populations employed during the research procedure from which it was normed. This would be particularly applicable in settings of high mental health comorbidity and state funded systems. Considering this, further research is required that can boast ecological validity, in order to establish the true usefulness of the ADOS for adulthood ASD diagnosis.

Indeed, the existence of comorbidity should be considered in relation to the low levels of specificity found here, as we know, high levels of comorbidity are reported in this population [4, 48–53]. As previously introduced, module four has had difficulty distinguishing between ASD and the other conditions, due to overlapping phenotypes with other conditions [10, 32].

Results from this study determined that scores on the ADOS-2 explained 25% of the variance in diagnostic outcome, accurately in 72.3% of cases, thus suggesting that other factors determine the remaining variance of the final outcome. This suggests there is value in a multidisciplinary team approach that can interpret the ADOS-2 scores rather than follow them without scrutiny. Whilst our results take a different approach towards exploring the usefulness of the ADOS assessment than previous studies, we make similar inferences as Molloy et al. [16] who found significant difference between multidisciplinary decision making on final diagnosis, compared to ADOS-2 outcome. The authors advise that there is sufficient evidence to deduce that if the ADOS-2 is employed as a strictly quantitative assessment, then it can lead to potential misclarification. Instead, ADOS-2 assessment may be most useful when considering the qualitative information gathered during the assessment.

The accuracy of the outcome of the ADOS-2 assessment is subjectively dependent on the team member who administers it. Whilst this should be taken into consideration, we believe that a particular strength of our study is that classification of outcome is formulated based on clinical judgements from highly experienced healthcare professions, from a multidisciplinary team. The ADOS-2 is a part of the process, but by no means determines the diagnostic outcome, as recommended by Lord et al. [18]. Another strength of our study was that all ADOS-2 assessment data were performed in a ‘real life’ clinical setting, by clinicians without knowledge that the data was going to be explored in this way. This gives an element of ecological validity compared to other studies. Based on this, we are confident in our diagnostic procedure and the importance of our results in developing this narrative.

## Conclusions

Understanding and diagnosing ASD in adulthood is under researched [2]. Studies such as ours are important in developing the narrative of issues specific to developing services for adults with ASD and also supporting clinicians to use their clinical judgement when making clinical decisions. The authors of the ADOS state that they did not intend for it to be used as a definitive diagnostic tool [18] and our finding support that assertion. The results from this study suggest that the results of the ADOS-2 module four should be interpreted with some caution if it is the only clinical evidence available. We would recommend based on our findings that clinicians reflect upon scores of standardised assessments with care, and not place particular emphasis on the numerical outcome. Instead, we recommend that diagnostic decisions are from an experienced multidisciplinary consensus of history taking, current observations, and qualitative information derived from assessments such as the ADOS-2, with particular interest paid to measures of restricted interests. What is required here is evidence-based, high-quality models of diagnostic assessment, without this, facilitating appropriate support and interventions is difficult for services. Future research should concern itself with this.

## Data Availability

The datasets used and/or analysed during the current study are available from the corresponding author on reasonable request.

## Abbreviations

ADHD: Attention deficit hyperactivity disorder
ADI-R: Autism diagnostic interview-revised
ADOS: The autism diagnostic observation schedule
ADOS-G: The autism diagnostic observation schedule generic
ADOS-2: The autism diagnostic observation schedule, second edition
ASD: Autism spectrum disorder
DSM-IV: Diagnostic and statistical manual of mental disorders, fourth edition
NHS: National health service
NICE: National institute for health and care excellence
NOS: Not otherwise specified
NPV: Negative predictive value
PDD: Pervasive developmental disorder
PPV: Positive predictive value
RRB: Restricted, repetitive behavior
ROC: Receiver operating characteristic curve
SD: Standard deviation
SWYPFT: South West Yorkshire partnership NHS foundation trust
